# New species of Brachystomellidae and characterization of *Micronella porcus* (Denis, 1933) from Brazil

**DOI:** 10.3897/zookeys.316.4869

**Published:** 2013-07-12

**Authors:** Gabriel C. Queiroz, Maria Cleide de Mendonça

**Affiliations:** 1 Departamento de Entomologia, Museu Nacional, Universidade Federal do Rio de Janeiro. Quinta da Boa Vista, São Cristóvão, 20.940-040 Rio de Janeiro-RJ, Brasil; 2 Doutorando do Programa de Pós-Graduação em Zoologia, Museu Nacional, Universidade Federal do Rio de Janeiro; 3 Professor Associado II do Museu Nacional, Universidade Federal do Rio de Janeiro

**Keywords:** Taxonomy, chaetotaxy, biodiversity, *Neorganella*, Neotropic

## Abstract

Three new species of Brachystomellidae from high altitude fields of southeast Brazil are described and illustrated and additions made to the description of *Micronella porcus* (Denis, 1933). The new species are *Neorganella rotundatae* **sp. n.**, the second for the genus, *Micronella itacaman* **sp. n.** and *Micronella longisensilla* **sp. n.** Diagnosis of the genera have been extended. An identification key to the genus *Micronella* Arlé, 1959 is provided.

## Introduction

The cosmopolitan family Brachystomellidae is, currently, comprised of 18 genera and 130 species (Bellinger et al. 2013). However, more than half (i.e. 10) the genera are monospecific and with restricted distributions.

The Neotropical fauna of Brachystomellidae is particularly diverse, especially for a group of euedaphic pale species that presents reductions of sense organs and appendages, such as eyes and/or furca. It is the case, for instance, of the Neotropical genera *Folsomiella* Bonet, 1930 (six species), *Maricaella* Mendonça & Fernandes, 1997 (monospecific), *Micronella* Arlé, 1959 (two species), *Neorganella* Rapoport & Rubio, 1963 (monospecific) and *Winterella* Massoud, 1967 (monospecific). The first three genera occurs in different habitats, such as sandy seashores and its surrounding vegetation, tropical forests and high altitude in mountains of the Andes, while *Neorganella* and *Winterella* are only found at high altitude mountains of the Andes (above 2,000 m a.s.l.).

In Brazil, the Brachystomellidae fauna comprises 19 species in seven genera (Abrantes et al. 2012). Of these, seven species belong to the group mentioned above: *Folsomiella albida* (Arlé, 1959), *Folsomiella caeca* (Folsom, 1927), *Folsomiella intermedia* (Arlé, 1939), *Folsomiella pseudocaeca* Mendonça et al., 2005, *Folsomiella trisetosa* Mendonça et al., 2005, *Maricaella duna* Mendonça & Fernandes, 1997 and *Micronella porcus* (Denis, 1933).

Recent expeditions, in order to sample the collembolan biodiversity from summits of three of the highest mountain plateaus of southeastern Brazil, always over than 2,000 m a.s.l., have revealed three new pale Brachystomellidae species which are herein described and illustrated: *Micronella itacaman* sp. n., *Micronella longisensilla* sp. n. and *Neorganella rotundatae* sp. n. In addition, a new record of *Micronella porcus* from the State of Minas Gerais, Brazil, and, due to its succinct original description, that lacks body chaetotaxy and other characters, these specimens are characterized and illustrated.

## Abbreviations used in text

Abd–abdominal segment;

Ant–antennal segment;

a.s.l.–above sea level;

Cx–coxa;

Fe–femur;

ICMBio–Instituto Chico Mendes da Biodiversidade;

MG–Minas Gerais State;

MNHN–Muséum National D'Histoire Naturelle;

MNRJ–Museu Nacional do Rio de Janeiro;

PAO–postantennal organ;

RJ–Rio de Janeiro State;

Scx–subcoxa;

Th–thoracic segment;

Tita–tibiotarsus;

Tr–trochanter.

## Remarks on *Micronella* and *Neorganella*

The genus *Micronella* was erected by Arlé (1959) in order to separate the species *Salmonella porcus* (Denis, 1933), originally described as a *Brachystomella*, from its congeners. Both *Micronella* Arlé, 1959 and *Setanodosa* Salmon, 1942 (*Salmonella* Stach 1949 was synonymized with *Setanodosa* by Massoud, 1967) are devoid of furca and the main difference between them is the absence of eyes and pigmentation of *Micronella*. Latter, a species from high altitude (2,400–4,200 m a.s.l.) in the Peruvian Andes, *Micronella checayensis* Winter, 1962 nom.nud., was validated by Massoud (1967), after examination of the type material.

**Table:** Table of localities <br/>

**Species**	**Latitude**	**Longitude**
*Micronella itacaman* sp. n.	22°22'59"S, 44°40'01"W	
	22°27'38"S, 43°01'45"W	
	20°26'07"S, 41°47'54"W	
*Micronella longisensilla* sp. n.	22°27'38"S, 43°01'45"W	
*Micronella porcus* (Denis, 1933)	20°26'07"S, 41°47'54"W	
*Neorganella rotundatae* sp. n.	22°22'59"S, 44°40'01"W	

Both *Micronella* species were briefly described, without any mention to dorsal body and also the furcal area chaetotaxy, which contains a set of chaetae that can be of taxonomic importance. Nevertheless, the analysis of Brachystomellidae made by Najt et al. (2005), which includes information on *Micronella porcus*, the following characterization of Brazilian specimens of *Micronella porcus* and of other two new species allow the expansion of the diagnosis of the genus.

Concerning *Neorganella* Rapoport & Rubio, 1963, the only species of the genus *Neorganella nothofagutalis* Rapoport & Rubio, 1963 was described based on a single specimen from a mountain called “El Roble”, of about 2,000 m a.s.l. and 50km from the Pacific Ocean. In 1967, Massoud synonymized *Neorganella* with *Folsomiella* and this remained until recently, when Najt et al. (2005), in an analysis of Brachystomellidae, revalidated the genus *Neorganella*.

As for the first two species of *Micronella*, there is no reference to head and most of the dorsal body chaetotaxy, regardless of the drawing of Abd III–VI in the original description, which is not elucidative. Nevertheless, the genus is well established among the Brachystomellidae, due to the presence of a reduced furca without mucro. The analysis of Najt et al. (2005) and the description of *Neorganella rotundatae* sp. n. supports the genus and allows the expansion of its diagnosis.

### Micronella

Arlé, 1959

http://species-id.net/wiki/Micronella

Brachystomella Syn. Agren, 1903 ad. part.
Salmonella Stach 1949 ad. part.

#### Type species.

*Brachystomella porcus* Denis, 1933.

**Diagnosis.** Pigmentation absent. Antennae shorter than head diagonal. Ant IV with dorsolateral microsensillum and round subapical organite; apical vesicle simple. Eyes absent. PAO with 6–15 vesicles. Maxilla typical of *Brachystomella*, with 5–7 teeth. Unguis tooth present or absent; tenent hair acuminated. Ventral tube with 3+3 chaetae. Furcal area delimited by a circular region of primary granulation of the tegument and a set of six chaetae within it. Table 1 summarizes the main characters of the species of the genus.

**Table 1.** Main characters of species of *Micronella* Arlé, 1959.<br/>

**Species**	***checayensis* Massoud, 1967**	***porcus* (Denis, 1933)**	***itacaman* sp. n.**	***longisensilla* sp. n.**
**Ant IV sensilla**	?	6	5	4
**Shape of sensilla of Ant III organ**	curved, opposite sense	“club”	one bilobed, one “club”	“cloverleaf”
**PAO vesicles**	15	6–8	7–8	12–13
**Ratio ordinary chaetae: sensilla**	?	1:1.3	1:1	1:2
**Serrated chaetae on body**	?	–	+	–
**Unguis tooth**	+	–	+†	–
**Type Locality**	Peru	Costa Rica	Brazil (southeast)	Brazil (RJ)

^†^Seen only on unguis of Tita I and II.

#### Key to the species of *Micronella* Arlé, 1959

**Table:** 

1	PAO with up to 13 vesicles; unguis without or with minute inner tooth	2
–	PAO with 15 vesicles, unguis with inner tooth	*Micronella checayensis* Massoud, 1967
2	PAO with up to 8 vesicles; ratio chaetae: sensilla approximatelly 1:1	3
–	PAO with 12–13 vesicles; ratio chaetae: sensilla = 1:2	*Micronella longisensilla* sp. n.
3	Ant IV with six sensilla; smooth chaetae on body; unguis without inner tooth	*Micronella porcus* (Denis, 1933)
–	Ant IV with five sensilla; serrated chaetae on body; unguis of Tita I and II with minute inner tooth	*Micronella itacaman* sp. n.

### Micronella itacaman sp. n.

urn:lsid:zoobank.org:act:E02CF5EA-2148-496C-BC05-253533ABFACB

http://species-id.net/wiki/Micronella_itacaman

Figs 1 –14

#### Type material.

Holotype: female, on slide. Label: Nº 2332 CM/MNRJ, Itatiaia, RJ, Brasil, Queiroz, G.C. leg, 27.iii.2012, 22°22'59"S, 44°40'1"W. Paratype: 1 female on slide, Label: Nº 2138 CM/MNRJ (D), Itatiaia, RJ, Brasil; Queiroz, G.C. leg, 14.vii.2011, 22°22'59"S, 44°40'1"W. Deposited at MNRJ, Rio de Janeiro, Brazil.

#### Type locality.

Brasil, Rio de Janeiro: Itatiaia municipality, Parque Nacional de Itatiaia (ICMBio), 22°22'59"S, 44°40'1"W, leaf litter and soil of “campos de altitude”, 2,400 m a.s.l.

**Other material.** One female on slide, Label: Nº 2153 CM/MNRJ (A), Alto Caparaó, MG, Brasil, Queiroz, G.C. leg, 27.vii.2011, 20°26'7"S, 41°47'54"W. Deposited at MNRJ, Rio de Janeiro, Brazil. One specimen deposited at MNHN, Paris, France: female, on slide, MNHN-EA011504, Teresópolis, RJ. Brasil, Queiroz, G.C. leg; 30.iii.2011, 22°27'38"S, 43°1'45"W.

#### Description.

Habitus typical of the genus. Body length of holotype: 0.88 mm; body length range of paratypes: 0.63–0.90 mm. Color in ethanol: white, no pigmentation.

**Figures 1–8.**
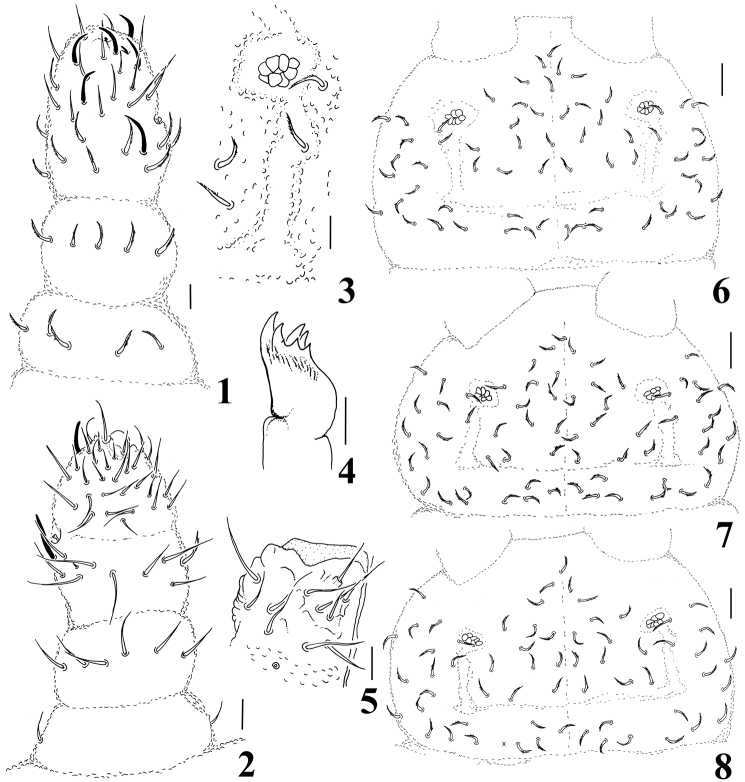
*Micronella itacaman* sp. n. **1** Dorsal view of Ant I–IV 2. Ventral view of Ant I–IV **3** PAO and its surrounding chaetae **4** Maxilla **5** Labium **6** Head chaetotaxy of specimen from Itatiaia **7** Head chaetotaxy of specimen from Teresópolis **8** Head chaetotaxy of specimen from Alto Caparaó. Scale bars: 10μm (**1–5**); 20 μm (**6–8**).

Ratio head diagonal: antenna = 1:0.63. Ant I with 7 chaetae. Ant II with 12 chaetae. Ant III and IV fused dorsally, ventral separation marked. Sensory organ of Ant III with two small club shaped sensilla, the mid-ventral one with a bilobed apex; two longer and subcylindrical guard sensilla; ventral microsensillum present (Figs 1–2). All dorsal chaetae of Ant I–III are serrated, ventral chaetae smooth and longer than those from dorsal side (10–13 μm dorsal; 13–15 μm ventral). Ant IV with simple apical bulb and five sensilla, three weakly differentiated from ordinary chaetae; dorsolateral microsensillum present; subapical organite round; with about 30 ventral chaetae (Fig. 2).

Without eyes. PAO bearing 7–8 vesicles disposed as a rosette (Fig. 3). Maxilla quadrangular with 6–7 teeth (Fig. 4). Labral formula: 2/2334. Labium typical of *Brachystomella*, with one papillated chaeta (L) and four proximal chaetae (Fig. 5).

**Figures 9–14.**
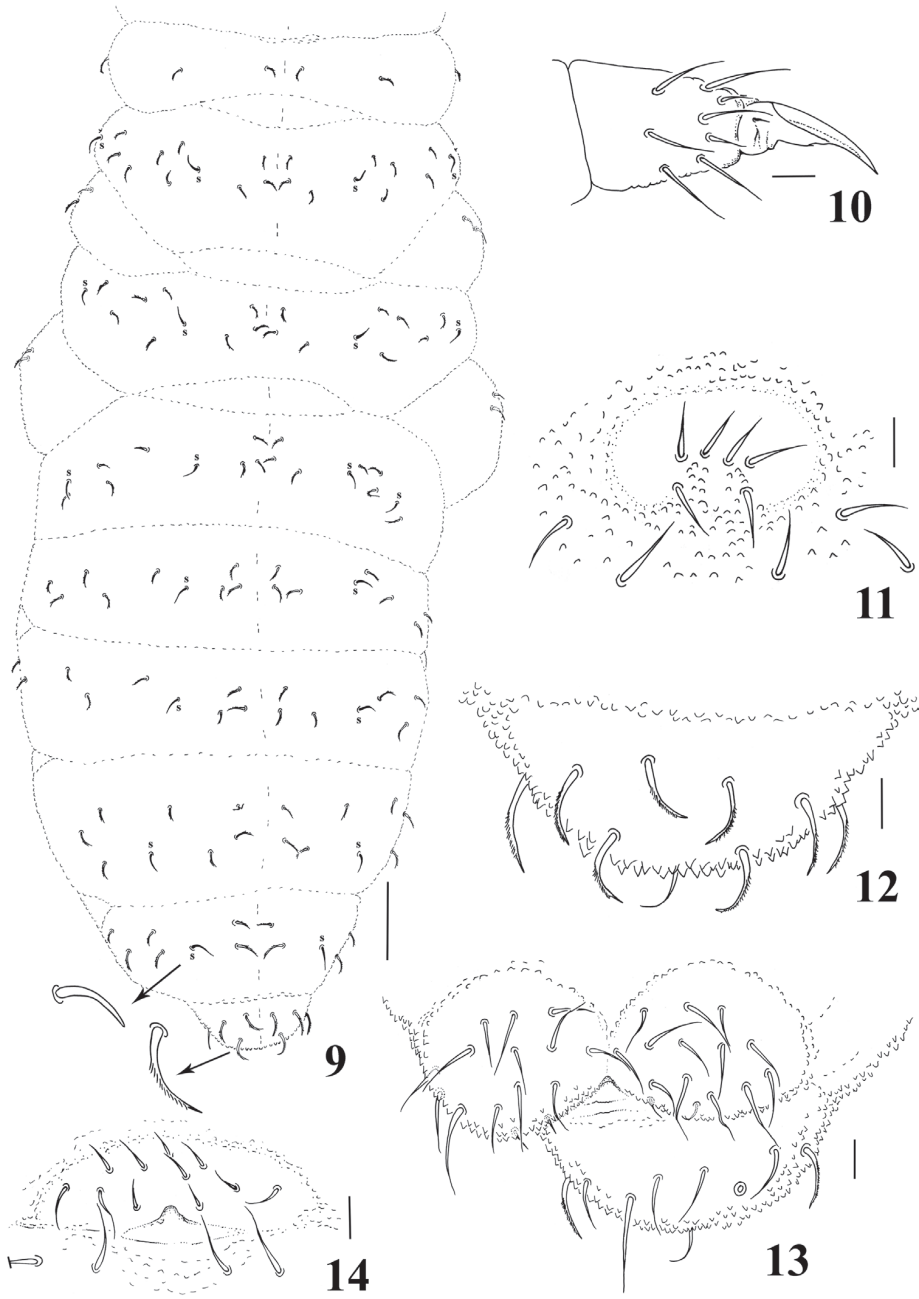
*Micronella itacaman* sp. n. 9. Dorsal body chaetotaxy with details of sensilla and chaetae **10** Tita of leg I **11** Furcal area **12** Dorsal view of Abd VI **13** Anal valves and ventral view of Abd VI **14** Female genital plate. Scale bars: 10μm (10–14); 50μm (9).

Head chaetotaxy as in Figs 6–8; asymmetries in the number of axial chaetae. Chaetae a0 present; Oc chaetae 3+3. Dorsal chaetotaxy composed of ordinary serrated chaetae and sensilla subequal in size, becoming longer towards the distal segments of the body (15 µm in Th I and 25 µm in Abd VI) (Fig. 9). Ratio of body ordinary chaetae: sensilla = 1:1. Th I with 2+2 chaetae; sensillar formula by half tergum: 022/211110.

Chaetotaxy of legs I–III as follows: Scx I– 1, 2, 2; Scx II– 0, 2, 2; Cx– 3, 6, 7; Tr– 5, 5, 5; Fe– 12, 10, 10; Tita– 19, 19, 18. All chaetae of Scx I of legs I–III are serrated. Tenent hair on tibiotarsi acuminate; unguis of legs I and II with one extremely minute median inner tooth; tooth not seen on unguis of leg III (Fig. 10). Ventral tube with 3+3 chaetae. Without tenaculum. Furca completely absent, but with a well-defined furcal area with six chaetae arranged in two rows: anterior row with four chaetae and posterior row with two chaetae (Fig. 11). Abd VI with 4+4 serrated chaetae and one unpaired smooth chaetae on dorsal side (Fig. 12). Each anal valve with 12–13 chaetae and 2 hr chaetae; Abd VI with 3+3 smooth chaetae on ventral side (Fig. 13). Female genital plate as in Fig. 14.

#### Etymology.

“Itakamã” (pronounced itakaman) means “high stone” or “rocky mountain” in the indigenous language Tupi, spoken by the Brazilian natives, reference to the three highest mountain plateaus of southeast Brazil, where the species was found.

#### Discussion.

The new species, *Micronella itacaman* sp. n., is well characterized in the genus, as all the species share euedaphic characters such as absence of eyes and furca, but with PAO. It can be distinguished from its congeners by characters such as serrated chaetae on body and five sensilla on Ant IV. In relation to number of vesicles on PAO and ratio of ordinary chaetae: sensilla, the new species is most similar to *Micronella porcus*, as they have 6–8 vesicles and a ratio of ordinary chaetae: sensilla of approximately 1:1.

### Micronella longisensilla sp. n.

urn:lsid:zoobank.org:act:B4515695-AD7E-4436-A3BF-FF131F3C9F76

http://species-id.net/wiki/Micronella_longisensilla

Figs 15 –28

#### Type material.

Holotype: female, on slide. Label: Nº 2207 CM/MNRJ (B), Teresópolis, RJ. Brasil, Queiroz, G.C. leg; 09.xi.2011, 22°27'38"S, 43°1'45"W. Paratypes: 4 females on slides and 1 specimen in ethanol, 2207 CM/MNRJ (A and C), same data as holotype. Deposited at MNRJ, Rio de Janeiro, Brazil.

#### Other material.

One female on slide. Label: Nº 2020 CM/MNRJ, Teresópolis, RJ. Brasil, Queiroz, G.C. leg; 30.iii.2011, 22°27'38"S, 43°1'45"W; 1 specimen in ethanol, 2023 CM/MNRJ, Teresópolis, RJ. Brasil, Queiroz, G.C. leg; 30.iii.2011, 22°27'38"S, 43°1'45"W; 2 females on slide, Nº 2092 CM/MNRJ (C), Teresópolis, RJ. Brasil, Queiroz, G.C. leg; 29.vi.2011, 22°27'38"S, 43°1'45"W; 1 female on slide and 3 specimens in ethanol, Nº 2211 CM/MNRJ (A), Teresópolis, RJ. Brasil, Queiroz, G.C. leg; 10.xi.2011, 22°27'38"S, 43°1'45"W; 1 female on slide, Label: Nº 2212 CM/MNRJ (D), Teresópolis, RJ. Brasil, Queiroz, G.C. leg; 10.xi.2012, 22°27'38"S, 43°1'45"W; 1 female on slide, Label: Nº 2302 CM/MNRJ (A), Teresópolis, RJ. Brasil, Queiroz, G.C. leg; 14.iii.2012, 22°27'38"S, 43°1'45"W; 1 specimen in ethanol, Nº 2307 CM/MNRJ, Teresópolis, RJ. Brasil, Queiroz, G.C. leg; 14.iii.2012, 22°27'38"S, 43°1'45"W; 1 specimen in ethanol, Nº 2314 CM/MNRJ, Teresópolis, RJ. Brasil, Queiroz, G.C. leg; 15.iii.2012, 22°27'38"S, 43°1'45"W; 1 specimen in ethanol, Nº 2317 CM/MNRJ, Teresópolis, RJ. Brasil, Queiroz, G.C. leg; 15.iii.2012, 22°27'38"S, 43°1'45"W. Deposited at MNRJ, Rio de Janeiro, Brazil. Two specimens deposited at MNHN, Paris, France: 1 female on slide, MNHN-EA011506, Teresópolis, RJ. Brasil, Queiroz, G.C. leg; 10.xi.2011, 22°27'38"S, 43°1'45"W; 1 female on slide, MNHN-EA011505, Teresópolis, RJ. Brasil, Queiroz, G.C. leg; 14.iii.2012, 22°27'38"S, 43°1'45"W.

#### Type locality.

Brasil, Rio de Janeiro, Teresópolis municipality, Parque Nacional da Serra dos Órgãos (ICMBio), 22°27'38"S, 43°1'45"W, leaf litter and soil of “campos de altitude”, 2,100 m a.s.l.

#### Description.

Habitus typical of the genus. Body length of holotype: 0.62 mm; body length range of paratypes: 0.40–0.75 mm. Color in ethanol: white, no pigmentation.

**Figures 15–21.**
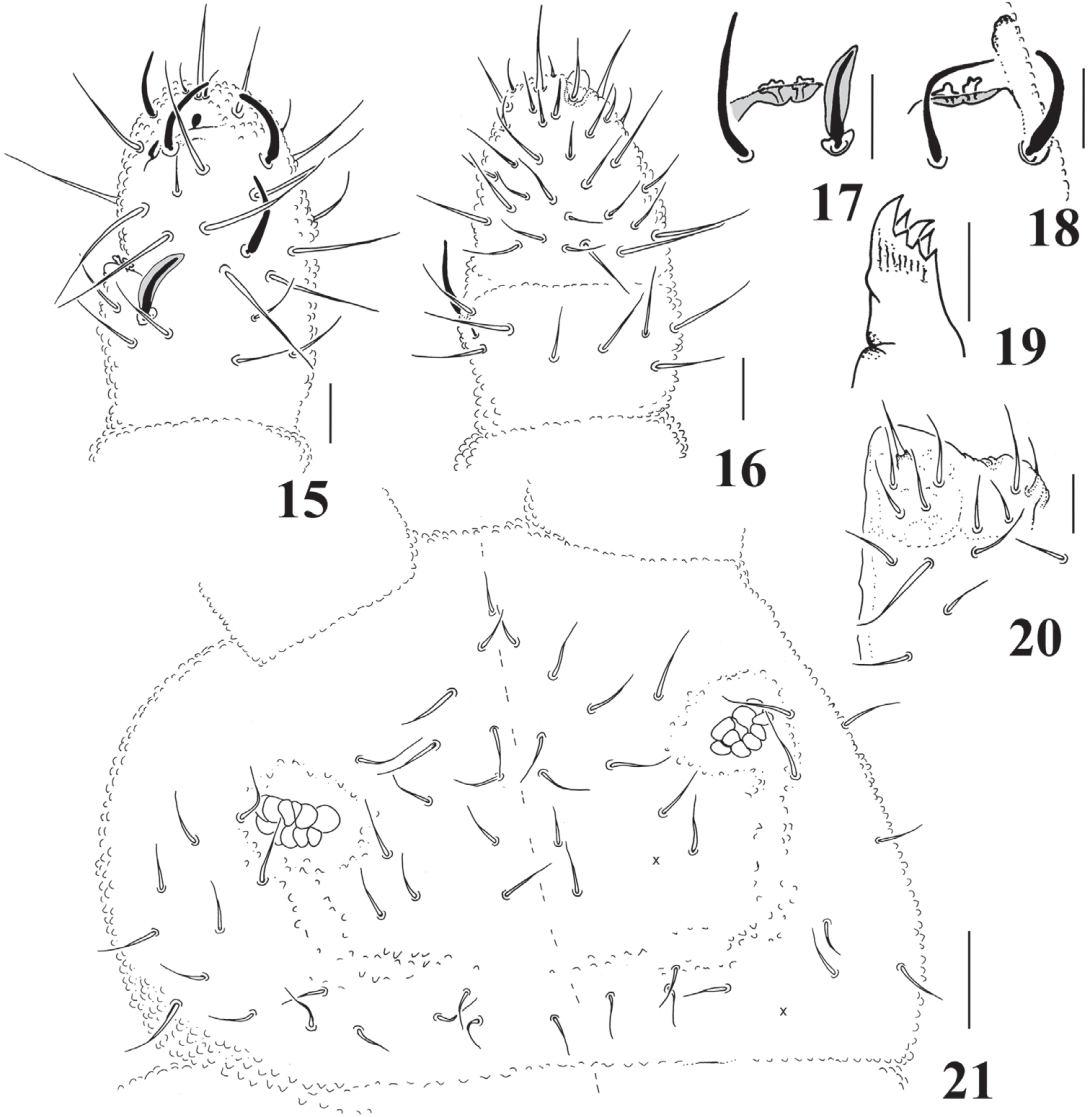
*Micronella longisensilla* sp. n. **15** Dorsal view of Ant III–IV **16** Ventral view of Ant III–IV **17** Detail of Ant III organ **18** Detail of Ant III organ (same specimen of Fig. 17, right antennae) **19** Maxilla **20** Labium **21** Head. Scale bars: 10μm (**15–20**); 20μm (**21**). x represents missing chaeta.

Ratio head diagonal: antenna = 1:0,.66. Ant I with 7 chaetae. Ant II with 11 chaetae. Ant III and IV fused dorsally, ventral separation marked. Sensory organ of Ant III with two cloverleaf-shaped sensilla partially covered by a fold of the integument; two longer and subcylindrical guard sensilla, the dorsal one is shorter but greatly enlarged in its width, in relation to the ventral one; ventral microsensillum present (Figs 15–18). Ant IV with simple apical bulb and four sensilla; dorsolateral microsensillum present; subapical organite round; with about 30 ventral chaetae (Fig. 16).

Without eyes. PAO bearing 12–13 vesicles disposed as a rosette. Maxillae quadrangular with 6–7 teeth (Fig. 19). Labral formula: 2/2334. Labium typical of *Brachystomella*, with one papillated chaeta (L) and four proximal chaetae (Fig. 20).

**Figures 22–28.**
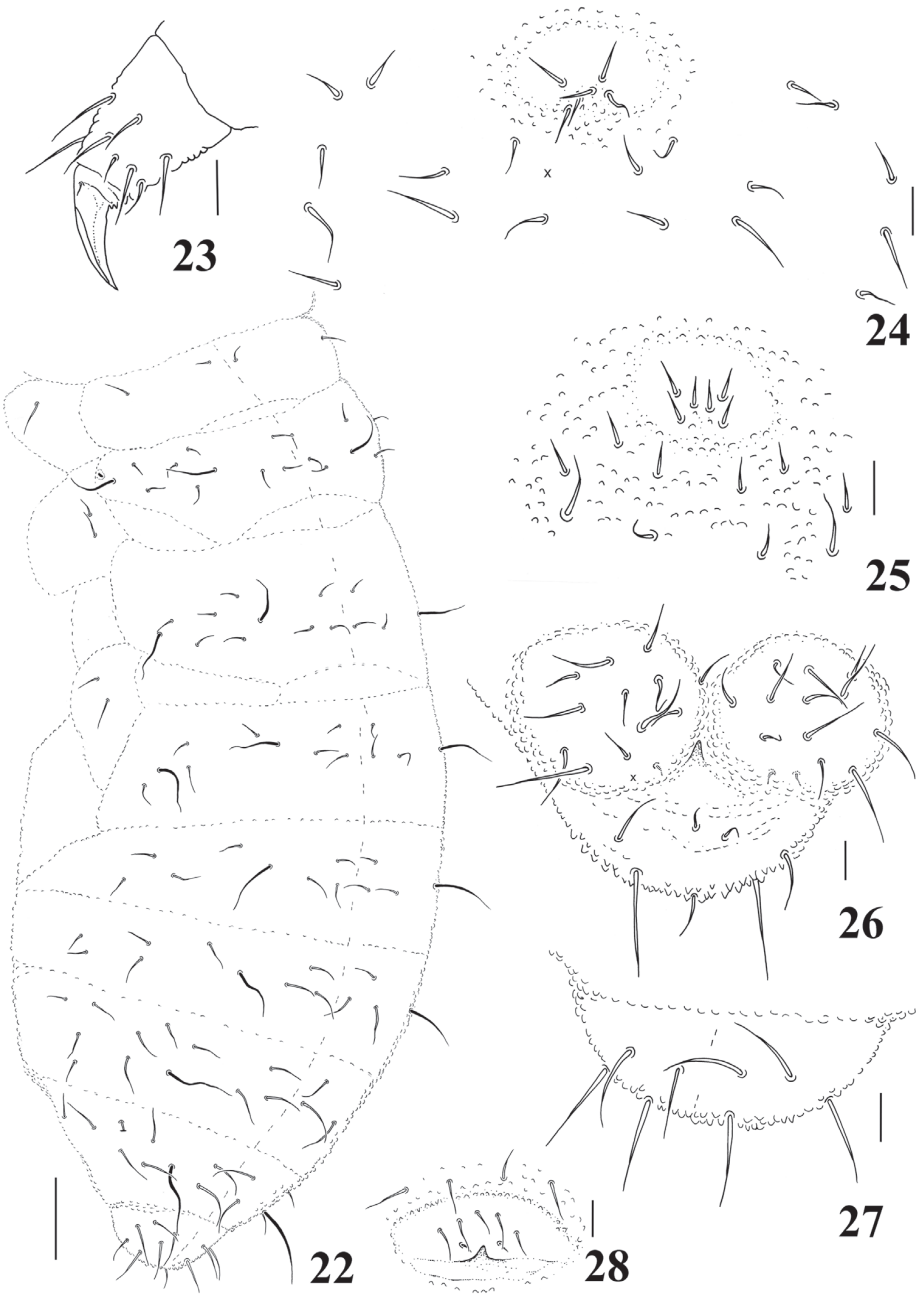
*Micronella longisensilla* sp. n. **22** Dorsolateral body chaetotaxy **23** Tita of leg I **24** Furcal area and its surrounding chaetae (adult) **25** Furcal area and its surrounding chaetae (juvenile) **26** Anal valves and ventral view of Abd VI **27** Dorsal view of Abd VI **28** Female genital plate. Scale bars: 10μm (**23–28**); 50μm (**22**). x represents missing chaeta.

Head chaetotaxy as in Fig. 21. Chaetae a0 present; Oc chaetae 3+3, sometimes asymmetric of 2+3. Dorsal chaetotaxy composed of smooth ordinary chaetae (10–25μm) and long sensilla (25–50μm), that becomes longer towards distal segments of the body. Ratio body ordinary chaetae: sensilla = 1:2. Th I with 2+2 chaetae; sensillar formula by half tergum: 022/211110 (Fig. 22).

Chaetotaxy of legs I–III as follows: Scx I– 1, 2, 2; Scx II– 0, 2, 2; Cx– 3, 6, 7; Tr– 5, 5, 4; Fe– 12, 11, 10; Tita– 19, 19, 18. Tenent hair on tibiotarsi acuminate; unguis without tooth (Fig. 23). Ventral tube with 3+3 chaetae. Without tenaculum. Furca completely absent, but with a well-defined furcal area with six chaetae (Figs 24–25).Each anal valve with 11–12 chaetae and 2 hr chaetae; Abd VI with 3+3 chaetae on ventral side, 4+4 chaetae on dorsal side and one unpaired chaetae (Figs 26–27). Female genital plate as in Fig. 28.

#### Etymology.

In a reference to the size of the sensilla in relation to ordinary chaetae on body of the new species.

#### Discussion.

The new species, *Micronella longisensilla* sp. n., is well characterized in the genus (see Table 1). It differs from its congeners in relation to the ratio of ordinary chaetae: sensilla, that is 1:2, only four sensilla on Ant IV, a PAO with 12–13 vesicles, the Ant III organ with two cloverleaf-shaped sensilla under a fold of the tegument and the dorsal guard sensilla which is greatly enlarged in its width, in relation to the ventral one.

### Brachystomella porcus

Denis, 1933

http://species-id.net/wiki/Brachystomella_porcus

Figs 29 –41

#### Examined material.

One female on slide, Label: Nº 2037 CM/MNRJ (C), Alto Caparaó, MG, Brasil, Queiroz, G.C. leg, 12.iv.2011, 20°26'7"S, 41°47'54"W; 1 female on slide and 2 specimens in ethanol, Nº 2041 CM/MNRJ (D), Alto Caparaó, MG, Brasil, Queiroz, G.C. leg, 13.iv.2011, 20°26'7"S, 41°47'54"W; 1 female and 1 juvenile on slides, Label: Nº 2353 CM/MNRJ (C and E), Alto Caparaó, MG, Brasil, Queiroz, G.C. leg, 11.iv.2012, 20°26'7"S, 41°47'54"W; 1 young female and 1 juvenile on slides, Label: Nº 2354 CM/MNRJ (A and B), Alto Caparaó, MG, Brasil, Queiroz, G.C. leg, 11.iv.2012, 20°26'7"S, 41°47'54"W. Deposited at MNRJ, Rio de Janeiro, Brazil. Two specimens deposited at MNHN, Paris, France: 1 female on slide MNHN-EA011501; 1 female on slide, Label: MNHN-EA011500, Alto Caparaó, MG, Brasil, Queiroz, G.C. leg, 13.iv.2011, 20°26'7"S, 41°47'54"W.

#### Locality.

Brasil, Minas Gerais: Alto Caparaó municipality, Parque Nacional do Caparaó (ICMBio), 20°26'7"S, 41°47'54"W, leaf litter and soil of “campos de altitude”, 2,700 m a.s.l.

#### Characterization of Brazilian specimens.

Habitus typical of the genus. Body length range of specimens: 0.45–0.95 mm. Color in ethanol: white, no pigmentation.

**Figures 29–34.**
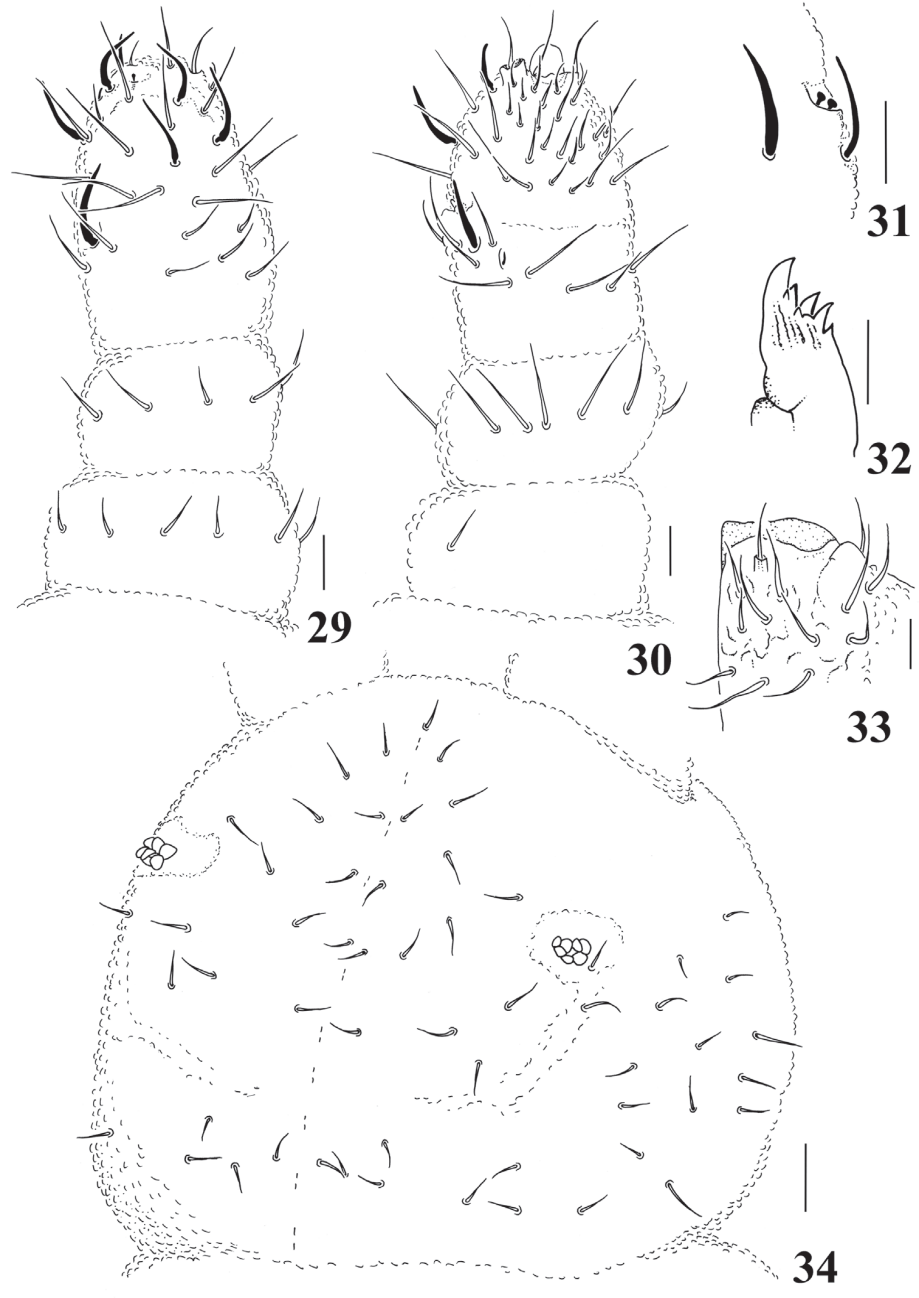
*Micronella porcus* (Denis, 1933). 29. Dorsal view of Ant I–IV **30** Ventral view of Ant I–IV **31** Detail of Ant III organ **32** Maxilla **33** Labium **34** Head chaetotaxy. Scale bars: 10μm (**29–33**); 20μm (**34**).

Ratio head diagonal: antenna = 1:0.57. Ant I with 7 chaetae. Ant II with 12 chaetae. Ant III and IV fused dorsally, ventral separation marked. Sensory organ of Ant III with two club-shaped sensilla; two longer and subcylindrical guard sensilla, the dorsal is stouter than ventral guard sensilla; ventral microsensillum present (Figs 29–31). Ant IV with simple apical bulb and six slender sensilla; dorsolateral microsensillum present; subapical organite round; with about 30 ventral chaetae (Fig. 30).

Without eyes. PAO bearing 7–8 vesicles disposed as a rosette. Maxilla quadrangular with 6–7 teeth (Fig. 32). Labral formula: 2/2334. Labium typical of *Brachystomella*, with one papillated chaeta (L) and four proximal chaetae (Fig. 33).

**Figures 35–41.**
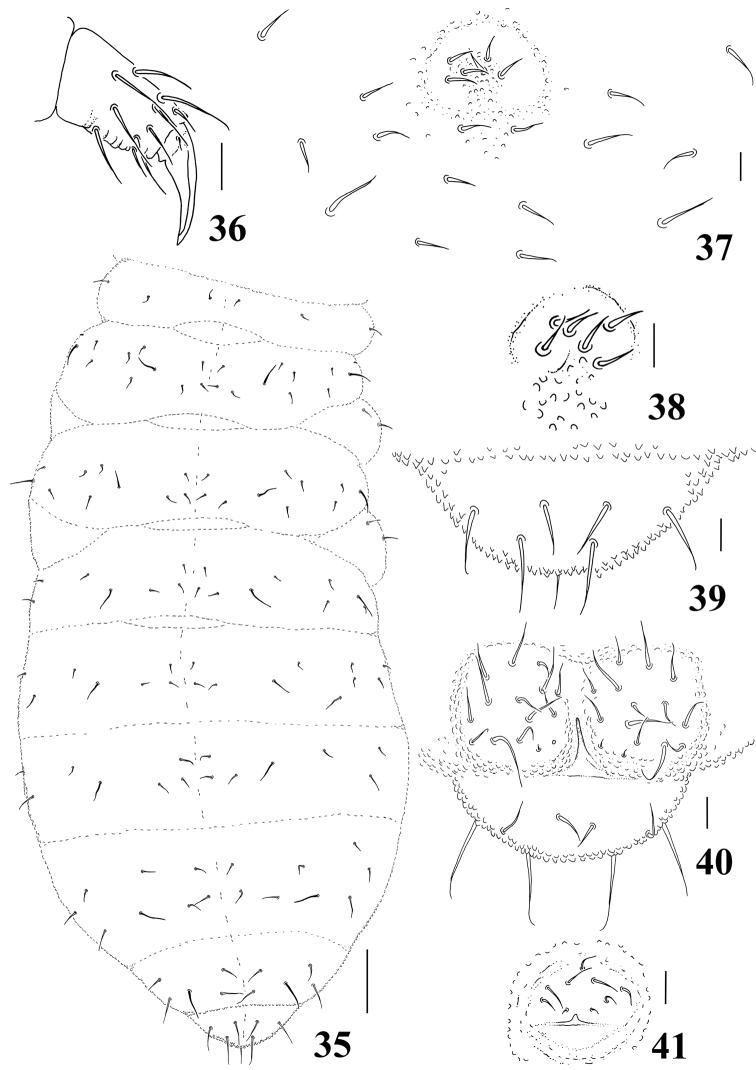
*Micronella porcus* (Denis, 1933). **35** Dorsal body chaetotaxy **36** Tita of leg I **37** Furcal area and its surrounding chaetae **38** Detail of furcal area **39** Dorsal view of Abd VI **40** Anal valves and ventral view of Abd VI **41** Female genital plate. Scale bars: 10μm (**36–41**); 50μm (**35**).

Head chaetotaxy as in Fig. 34. Chaetae a0 present, but some specimens with asymmetries; Oc chaetae 3+3. Dorsal chaetotaxy composed of smooth ordinary chaetae (15–20 µm) and longer sensilla (20–25 µm) that becomes longer towards distal segments of the body. Ratio ordinary chaetae: sensilla = 1:1.3. Th I with 2+2 chaetae; sensillar formula by half tergum: 022/211110 (Fig. 35).

Chaetotaxy of legs I–III as follows: Scx I– 1, 2, 2; Scx II– 0, 2, 2; Cx– 3, 6, 7; Tr– 5, 5, 4; Fe– 12, 11, 10; Tita– 19, 19, 18. Tenent hair on tibiotarsi acuminated; unguis without tooth (Fig. 36). Ventral tube with 3+3 chaetae. Without tenaculum. Furca completely absent, but with a well-defined furcal area with six chaetae (Figs 37–38). Abd VI with 4+4 chaetae on dorsal side and one unpaired chaetae; with 3+3 chaetae on the ventral side (Fig. 39). Each anal valve with 12–13 chaetae and 2 hr chaetae; (Fig. 40). Female genital plate as in Fig. 41.

#### Remarks.

The examined specimens from Minas Gerais State, Brazil, fit the description of the Neotropical species *Micronella porcus*. The six sensilla on Ant IV, the club-shaped sensilla on Ant III organ, the 6–8 vesicles on PAO and the toothless unguis are the main characters that define the species. The description above adds important characters such as head and dorsal body chaetotaxy and also the number of chaetae on furcal area to the original description.

### Neorganella

Rapoport & Rubio, 1963

http://species-id.net/wiki/Neorganella

#### Diagnosis.

Pigmentation absent, pale aspect. Antennae shorter than head diagonal. Ant IV with dorsolateral microsensillum and round subapical organite; apical vesicle simple. Eyes absent. PAO with 4–12 vesicles. Maxilla typical of *Brachystomella*, with 5–7 teeth. Unguis tooth present or absent; tenent hair acuminate. Ventral tube with 3+3 chaetae. Tenaculum present. Reduced furca: without mucro, but with two small rounded or globular dens, each with 3–4 chaetae.

### Neorganella rotundatae sp. n.

urn:lsid:zoobank.org:act:0AA68A6D-A503-4695-89E2-64893DEE33B3

http://species-id.net/wiki/Neorganella_rotundatae

Figs 42 –55

#### Type material.

Holotype: male, on slide, Label: Nº 1984 CM/MNRJ, Itatiaia, RJ, Brasil, Queiroz, G.C. leg, 14.iii.2011, 22°22'59"S, 44°40'1"W. Paratypes: 1 female and 4 juveniles on slides, Label: Nº 2133 CM/MNRJ (C and D), Itatiaia, RJ, Brasil, Queiroz, G.C. leg, 13.vii.2011, 22°22'59"S, 44°40'1"W. Deposited at MNRJ, Rio de Janeiro, Brazil. Two specimens deposited at MNHN, Paris, France: 1 female on slide, MNHN-EA011502, Itatiaia, RJ, Brasil, Queiroz, G.C. leg, 13.vii.2011, 22°22'59"S, 44°40'1"W1 juvenile on slide, MNHN-EA011503, Itatiaia, RJ, Brasil, Queiroz, G.C. leg, 25.x.2011, 22°22'59"S, 44°40'1"W.

#### Type locality.

Brasil, Rio de Janeiro: Itatiaia municipality, Parque Nacional de Itatiaia (ICMBio), 22°22'59"S, 44°40'1"W, leaf litter and soil of “campos de altitude”, 2,400 m a.s.l.

#### Description.

Habitus typical of the family. Body length of holotype: 0.88 mm; body length range of paratypes: 0.47–1.20 mm. Color in ethanol: white, no pigmentation.

**Figures 42–47.**
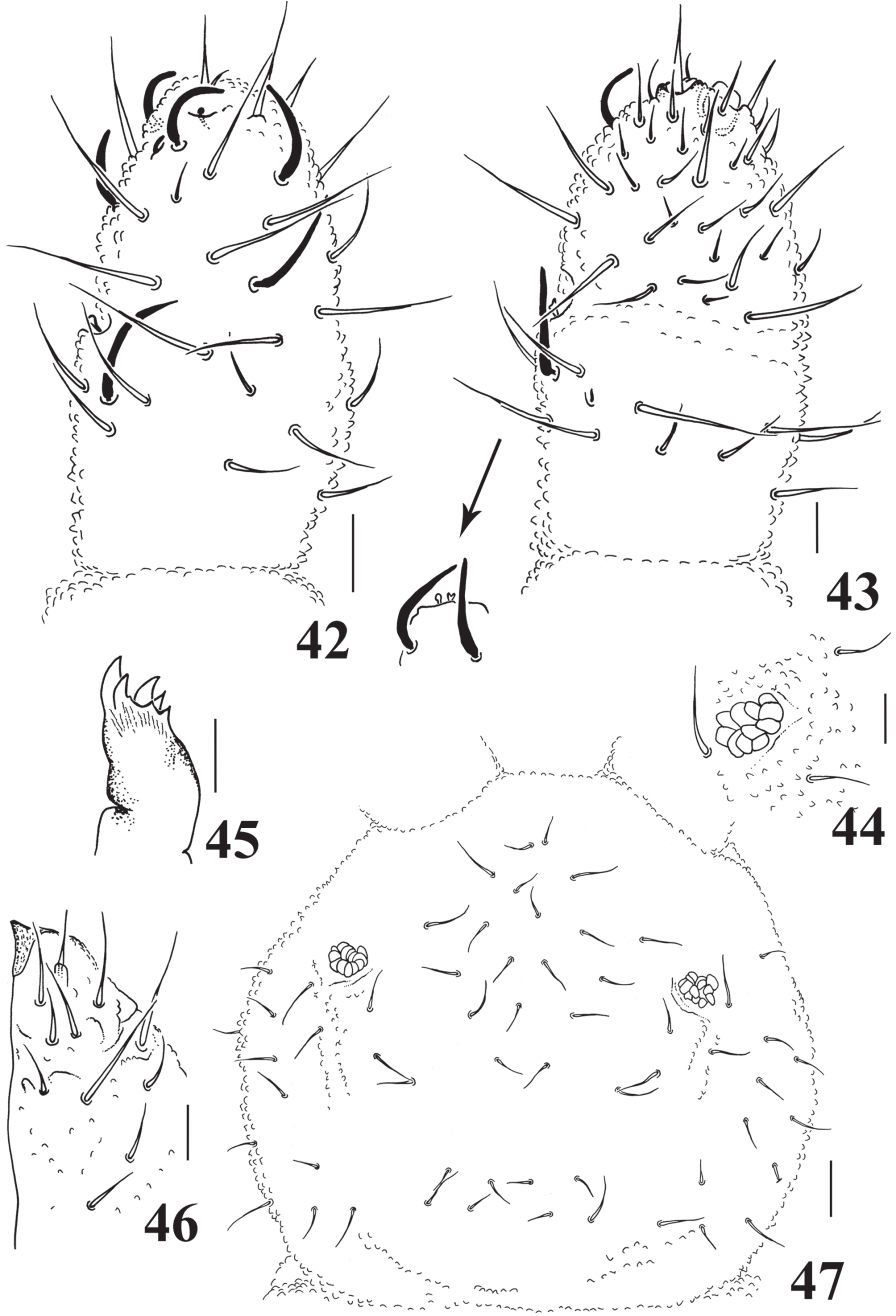
*Neorganella rotundatae* sp. n. **42** Dorsal view of Ant II–IV **43** Ventral view of Ant III–IV with detail of Ant III organ **44** Detail of PAO **45** Maxilla **46** Labium **47** Head chaetotaxy. Scale bars: 10μm (**42–46**); 20μm (**47**).

Ratio head diagonal: antenna = 1:0.63. Ant I with 7–8 chaetae. Ant II with 12 chaetae. Ant III and IV fused dorsally, ventral separation marked. Sensory organ of Ant III with two small club-shaped sensilla, the mid-ventral one with a bilobed apex; two longer and subcylindrical guard sensilla; ventral microsensillum present (Figs 42–43). Ant IV with simple apical bulb and five sensilla; dorsolateral microsensillum present; subapical organite round; about 30 ventral chaetae (Figs 42–43).

Without eyes. PAO bearing 10–12 vesicles disposed as an elongated rosette (Fig. 44). Maxilla quadrangular with 6–7 teeth (Fig. 45). Labral formula: 2/2334. Labium typical of *Brachystomella*, with one papillated chaetae (L) and four proximal chaetae (Fig. 46).

**Figures 48–55.**
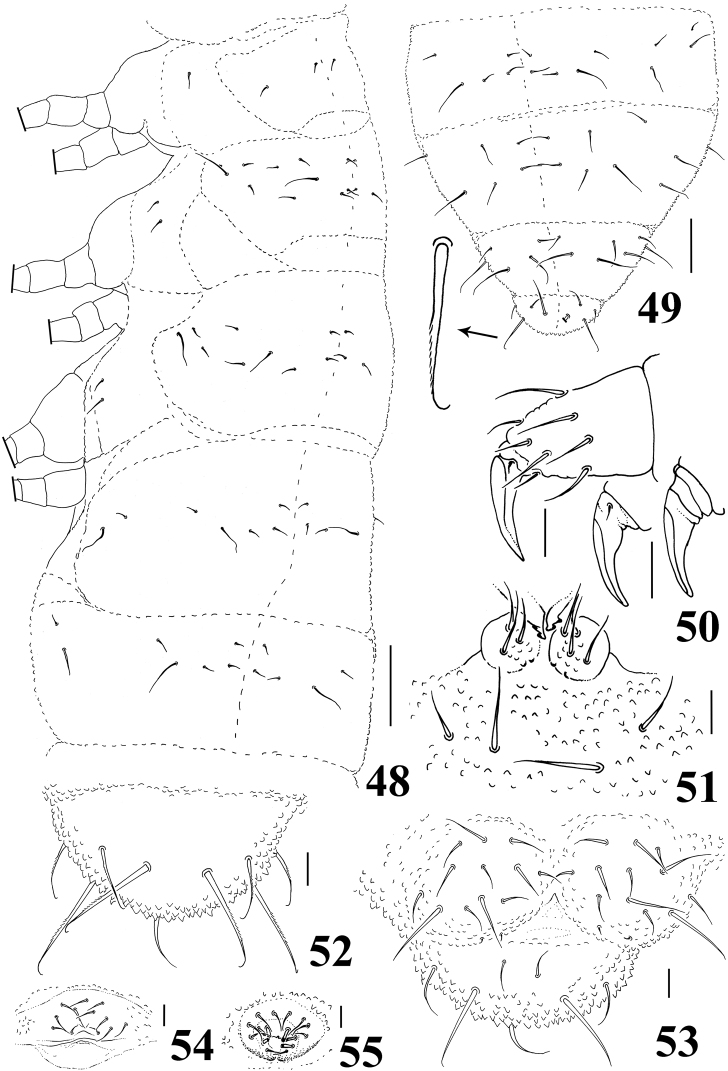
*Neorganella rotundatae* sp. n. **48** Dorsolateral chaetotaxy of Th I–Abd II **49** Dorsolateral chaetotaxy of Abd III–VI with detail of chaetae **50** Tita of leg II with detail of two unguis (left: unguis III; right: unguis II) **51** Tenaculum and reduced furca **52** Dorsal view of Abd VI **53** Anal valves and ventral view of Abd VI **54** Female genital plate **55** Male genital plate. Scale bars: 10μm (**50–55**); 50μm (**48–49**).

Head chaetotaxy as in Fig. 47. Chaetae a0 absent; Oc chaetae 3+3. Dorsal chaetotaxy composed of slightly serrated chaetae and longer sensilla (Fig. 48); Abd V with some longer chaetae, subequal to sensilla, and Abd VI with 4+4 serrated chaetae with a tendency to have bent tips (Fig. 49). Th I with 2+2 chaetae; sensillar formula by half tergum: 022/211110. All dorsal and lateral chaetae are slightly serrated.

Chaetotaxy of legs I–III as follows: Scx I – 1, 2, 2; Scx II – 0, 2, 2; Cx – 3, 6, 7; Tr – 5, 5, 5; Fe – 12, 12?, 10; Tita – 18, 18, 17. Tenent hair on tibiotarsi acuminated; unguis of legs I and II with one extremely minute median inner tooth; tooth not seen on unguis of leg III (Fig. 50). Ventral tube with 3+3 chaetae. Tenaculum small with 2 teeth on each ramus. Furca reduced to two small globular dens with 3–4 chaetae on each side and without mucro (Fig. 51). Abd VI with 4+4 serrated chaetae with bent tips, of which 2+2 are longer than others (25μm to 20μm), and one unpaired smooth chaetae on dorsal side (Fig. 52). Each anal valve with 12 chaetae and 2 hr chaetae; Abd VI with 3+3 smooth chaetae on ventral side (Fig. 53). Female and male genital plate as in Figs 54 and 55, respectively.

#### Etymology.

The Latin word *rotundatae* means roundish, spherical, referring to dens shape of the new species.

#### Discussion.

The new species *Neorganella rotundatae* sp. n. is well characterized in the genus, mainly due to the facts that it shares a reduced furca without mucro, dens with 3+3 chaetae, and the presence of tenaculum with the other species *Neorganella nothofagutalis* Rapoport & Rubio, 1963 (according to original description and after Najt et al. 2005). The new species differs from its congener by the presence of 10–12 vesicles on PAO, while *Neorganella nothofagutalis* has only 4 vesicles. It is also noteworthy that *Neorganella rotundatae* sp. n. presents a reduction in the number of chaetae on Tita of legs I–III, being 18, 18, 17, respectively, while *Neorganella nothofagutalis* has 19, 19, 18 (see Najt et al. 2005).

## Supplementary Material

XML Treatment for Micronella

XML Treatment for Micronella itacaman

XML Treatment for Micronella longisensilla

XML Treatment for Brachystomella porcus

XML Treatment for Neorganella

XML Treatment for Neorganella rotundatae
